# AXL is a novel ERK5/KLF4 target in MEK inhibitor-treated melanoma

**DOI:** 10.1016/j.neo.2026.101301

**Published:** 2026-03-30

**Authors:** Rupesh Paudel, Simon Goller, Stefanie Schwarz, Katharina Meder, Matthias Goebeler, Marc Schmidt

**Affiliations:** Department of Dermatology, Venereology and Allergology, University Hospital Würzburg, Josef-Schneider-Str. 2, Würzburg 97080, Germany

**Keywords:** NRAS-mutant melanoma, Therapy resistance, MEK5/ERK5 pathway, AXL, KLF2, KLF4

## Abstract

RAS/RAF/MEK/ERK1/2 mitogen-activated protein kinase (MAPK) pathway-based treatments, typically combination therapies with inhibitors for oncogenic BRAF V600 (BRAFi) and MEK1/2 (MEKi), are important treatment alternatives to immunotherapy in advanced *BRAF*-mutated melanoma. However, their benefit is limited by frequent therapy resistance, i.e. persistence and progression/metastasis of tumor cells under sustained treatment. Furthermore, approximately 50% of the patients, including the *NRAS*-mutated subset, lack targetable BRAF oncogenes and profit poorly from MEKi. Recent preclinical studies suggest co-inhibition of the MEK5/ERK5 MAPK pathway, which in different MAPK-activated tumors is compensatorily activated by MEKi, as promising strategy to overcome therapy resistance and trigger apoptosis and/or sustained cell cycle arrest. In *NRAS*-mutant melanoma, compensatory ERK5 activation is accompanied by the induction of the Krüppel-like factors KLF2 and KLF4 but their role in MEKi resistance remains unclear. Using RNA interference and CRISPR/*Cas9*, we examined their contribution to MEKi resistance through RNA sequencing and functional assays. Surprisingly, KLF2 and KLF4 were dispensable for the proliferative and anti-apoptotic effects of compensatory ERK5 activation in MEKi-exposed melanoma. Instead, we identified AXL, a key receptor tyrosine kinase associated with metastasis and phenotypic switching, as critical ERK5/KLF4 target induced during MEKi resistance. Genetic loss of KLF4 or AXL depletion reduced melanoma cell migration and invasion, suggesting a key role of KLF4 in the regulation of invasiveness. Our study describes a novel ERK5/KLF4/AXL signaling axis that drives MEKi resistance and metastatic potential in *NRAS*-mutant melanoma and highlights this axis as a potential target to improve MAPK-directed and potentially immune therapies.

## Highlights

• AXL is a key effector of the MEK5/ERK5/KLF4 pathway in melanoma.
• MEK5/ERK5 signalling drives a drug-resistant, AXL-high melanoma state.
• ERK5/KLF4 deficiency prevents MEKi-resistance-associated AXL induction.
• KLF4 depletion triggers rapid AXL loss in MEKi-resistant melanoma.
• The KLF4-AXL axis promotes melanoma cell migration and invasiveness.

**Abbreviations**
7AAD: 7-amino-actinomycin D
ANOVA: analysis of variance
DAVID: database for annotation, visualization, and integrated discovery
CRISPR: clustered regularly interspaced short palindromic repeats
*Cas9*: CRISPR-associated protein 9
DEG: differentially-expressed gene
ERK5i: ERK5 inhibition
EV: empty vector
FCS: fetal calf serum
KLF: Krüppel-like factor
MAPK: mitogen-activated protein kinase
MAPKi: mitogen-activated protein kinase pathway inhibition
MEKi: MEK inhibition
MFI: median fluorescence intensity
RNA-seq: RNA-sequencing
siScr: siRNA-scrambled
SCC: single cell clone
SD: standard deviation
Tram: trametinib
Tram^R^: trametinib-resistant

## Introduction

Over the past decade treatment of advanced malignant melanoma has made tremendous progress but its long-term success is diminished by the extraordinary versatility of melanoma to develop therapy resistance [1]. The latter is a gradual process, during which early persisting tumor cells progress under therapy by restarting the cell cycle and/or switching to a slow-cycling, immune-suppressive, therapy-tolerant, and metastatic state [2]. Notably, therapy resistance is both a problem with immunotherapy and targeted therapy aiming to interfere with the RAS/RAF/MEK/ERK1/2 mitogen-activated protein kinase (MAPK) pathway (MAPKi) that is predominantly constitutively active in cutaneous melanoma [3]. Efforts in melanoma therapy thus no longer solely focus on development of new therapeutics but also concentrate on the refinement of existing therapies by implementing strategies to prevent therapy resistance [1].

The stress-activated MEK5/ERK5 MAPK pathway has recently been identified as interesting therapeutic target, which co-inhibition was able to prevent therapy resistance to targeted MAPKi in several types of cancer, including *BRAF-* and *NRAS*-mutant cutaneous melanoma [[4], [5], [6], [7], [8]], *KRAS*-mutant colorectal [9], non-small cell lung [10] and pancreatic cancer [11], as well as HER2-positive breast cancer [12]. In all these tumors, inhibition of MEK, e.g. by exposure with the clinically approved MEK inhibitor (MEKi) Trametinib (Tram), triggered compensatory MEK5/ERK5 pathway activation, allowing tumor cells to persist and proliferate under targeted MEKi therapy [13]. In the case of *NRAS* mutant melanoma, MEKi-induced ERK5 pathway activation also extends to the transcriptional induction of the two Krüppel-like factors KLF2 and KLF4 [4]. These zinc-finger transcription factors are prominent stem cell factors that, together with OCT4, SOX2, and cMYC, redundantly enable the reprogramming of somatic cells into induced pluripotent stem cells [14]. However, depending on the context, they also control diverse differentiation and inflammation processes, proliferation and cellular survival [[15], [16], [17], [18]]. For instance, KLF4 was shown to regulate proliferation and cellular senescence by acting as a context-specific oncogene or tumor suppressor via regulating p53 expression [19]. Additionally, both KLFs represent established downstream effectors of the MEK5/ERK5 pathway in endothelial cells where they are involved in the regulation of migration, immune activation and cellular survival [13,[20], [21], [22]]. Elevated expression and/or nuclear localization of KLF2 or KLF4 is also increasingly recognized as an indicator of ERK5 activity in other cellular settings [4,[23], [24], [25], [26], [27]]. However, apart from their well-studied role in vascular cells, the precise function of KLF2 and KLF4 downstream of ERK5, particularly in the context of tumor resistance, remains unclear.

Here, we performed bulk RNA sequencing and functional assays to study the consequences of combined *KLF2* and *KLF4* knockdown or *MEK5* depletion on the MEKi-induced resistance of *NRAS*-mutant melanoma cells and clarify their role in ERK5-dependent therapy resistance. Our study reveals an unexpected role of ERK5 and KLF4 in the regulation of the pro-metastatic receptor tyrosine kinase AXL, which induction is closely linked to melanoma switching and acquisition of a therapy-resistant, invasive state.

## Materials and methods

### Cell culture, treatments and generation of tram-resistant cell lines

The *NRAS*-mutant melanoma cell lines FM79, BLM, M26, and MaMel26a and the *BRAF*-mutant melanoma cell lines A375 and FM88 were grown at 37°C with 5% CO_2_ in RPMI1640 GlutaMAX medium (Thermo Fisher Scientific, Darmstadt, Germany), supplemented with 10% fetal calf serum (FCS) (Capricorn, Ebsdorfergrund, Germany) and 30 µg/ml Gentamycin (#G1397, Sigma Aldrich, Darmstadt, Germany). Each cell line was routinely thawed freshly from frozen stocks stored at the Department of Dermatology, University Hospital, Würzburg, Germany, tested for mycoplasma positivity, and discarded after 10-15 passages. The established MEKi Trametinib (Tram) was purchased from Enzo (#ENZ-CHM239; Lörrach, Germany) and used at 5 nM (FM79) or 25 nM (BLM), respectively. The ERK5 inhibitors XMD8-92 (#HY-14443), JWG-071 (#HY-108886) and AX15836 (#HY-101846) were purchased from MedChemTronica (Sollentuna, Sweden), and used at 5 µM. Tram-resistant (Tram^R^) FM79 and BLM cells were independently established for each experiment by culturing drug-naïve cells in Tram-containing medium for a period of two weeks with regular media replacements every 3-4 days to prevent dominance of a single resistance mechanism. For short-term co-treatments of naïve and Tram^R^ cells, FM79 cells were seeded into Tram-free or Tram-containing growth media at a density of 1.25 × 10^4^ cells/cm^2^ and the following day exposed to medium containing Tram alone or Tram and 5 µM ERK5i for two days.

### Generation of *MEK5, MAPK7,* and *KLF4* -deficient cell lines by lentiviral CRISPR/*Cas9*

*MEK5* knockout BLM single-cell clones have been described earlier [7]. For generation of empty vector, scrambled (Scr), *MAPK7 (=ERK5),* and *KLF4* k.o. FM79 cells, a previously described lentiviral expression vector, lentiCRISPR_Zeo, was used [7] that encodes the respective guide (g) RNA and *Cas9* on a single plasmid. The respective gRNA sequences (Supp. Table 1) were either obtained from the Human CRISPR Knockout Pooled [28] libraries (gScr and g*ERK5*) or self-designed for g*KLF4* using the Chop-Chop database [29], and inserted into lentiCRISPR_Zeo vector by Golden Gate cloning. In case of KLF4, pan-specific gRNAs targeting all known splice variants including KLF4α, a recently described alternative splice variant with proposed oncogenic function [30,31], were designed to prevent misinterpretation due to exclusive disruption of single variants. Target FM79 cells were lentivirally infected as described [7], reseeded, and after positive selection with 400 µg/ml Zeocin (InvivoGen, Toulouse, France) used as polyclonal cell lines.

### siRNA-mediated gene knockdown

For knockdown experiments, the respective naïve or Tram-pre-exposed cell lines were transfected with either scrambled siRNA (siScr) or different siRNAs targeting the genes of interest as described [4]. Target sequences and catalogue numbers of the employed siRNAs are listed in Supp. Table 2. Depending on the experiment, siRNA-transfected cells were harvested directly 2 to 3 days after siRNA transfection (for RNA and protein isolation) or were trypsinized, counted and reseeded at equal density 24h post transfection for further processing (for proliferation and viability assays).

### Cellular proliferation and viability assay

Cellular proliferation and viability were determined by crystal-violet staining or flow-cytometric analysis of 7-amino-actinomycin D (7AAD) /annexin V positivity as described [4]. After siRNA-transfection, the different siRNA-manipulated BLM and FM79 cells were seeded in duplicate at a density of 8 × 10^4^ or 1 × 10^5^ cells /cm^2^ and one dish treated with vehicle, the other one exposed to Tram at 25 (BLM) or 5 nM (FM79), respectively. For crystal violet assays, culture media were replaced every 3-4 days, and cells stained after 7-10 days when vehicle controls reached confluency. For the 7AAD/Annexin-V assays, cells were treated with Tram or medium for 72h and viable attached cells and supernatants containing floating dead cells were pooled for harvesting. Subsequent flow cytometric stainings were performed by 7AAD/Annexin-V staining as described [4].

### AXL-cell surface staining

At the end of the respective experiment treatment-naïve or Tram^R^ melanoma cells were detached by brief incubation in accutase and stained with a polyclonal anti-AXL antibody (R&D System, AF154) according to manufacturer’s protocol. Staining with a suitable control antiserum served as background control.

### RNA isolation, cDNA synthesis and qRT-PCR

Total RNA was isolated, and equal amounts of RNA was transcribed to cDNA followed by SYBR-green-based qRT-PCR using gene-specific qRT-PCR primer sequences (Supp. Table 3) were performed as described previously [7] using an AriaMx cycler (Agilent, Waldbronn, Germany).

### Western blot

Western blots were performed as described [23] using the dilution and antibodies listed in Supp. Table 4. Western-blot intensities were quantified by densitometric analysis, as previously described [4]. Presented data each represent the ratio of the target protein value to that of the corresponding loading control protein after background correction. For better comparability, values were each normalized to the respective experimental control, which was arbitrarily set to 1.

### RNA-seq and bioinformatic analysis

FM79 cells were in parallel transfected with a scrambled siRNA (siScr), an siRNA against *MEK5*, or pools of each two siRNAs directed against *KLF2* and pan*KLF4*. The following day, cells were exposed to 5 nM Tram (all conditions) or medium (siScr control only) for 48h and processed for RNA isolation. A small aliquot of each RNA sample was used for RNA quantification, quality control (Bioanalyzer 2100, Agilent, Waldbrunn, Germany), and validation of proper knockdown efficiency by qPCR. For each condition RNA isolates of three independent biological replicates were then subjected to bulk RNA sequencing and bioinformatic analysis as described previously [7].

### Migration and invasion assays

To study the migration and invasion potential of the different genetically manipulated and treated BLM and FM79 cell lines, 5 × 10^3^/cm^2^ BLM cells or 1.25 × 10^4^ cells/cm^2^ FM79 were seeded into FCS-reduced media containing 0.5% FCS with or without Tram and incubated for 24h. Transwell TC inserts (Sarstedt #83.3932.800) or Corning BioCoat Matrigel chambers (Corning #354480) with 8 µM pore size were used for migration or invasion assays, respectively. Inserts were placed on a 24-well plate and equilibrated by addition of 250 µl of media containing 0.5% FCS for 10 minutes before aspirating it out. Cells were detached using accutase, counted, and 2 × 10^4^ cells reseeded again into FCS-reduced medium. Chemotaxis was initiated by adding medium containing 10% FCS in the respective wells of 24-well plates. After incubation for 24h, non-migrating or -invading cells remaining on the upper surface of the membranes were removed using a cotton tip and membranes fixed by incubation in 4% paraformaldehyde for 15 min and ice-cold methanol for 20 min. Subsequently, cells were stained with crystal-violet solution (0.25% w/v in 20% methanol v/v) for 1h and washed three times with tap water. Membranes were then cut out and embedded on slides with hydrophobic Entellan^TM^ mounting medium. For quantification, tile-scan brightfield photographs were made using a Nikon Ti motorized-stage microscope with 10x objective and five visual fields per condition counted manually. Finally, counts were normalized to an appropriate experimental control (set to 1) prior to statistical evaluation of multiple independent experiments.

### Statistical analysis

All experiments were reproduced at least twice unless stated otherwise. Data were statistically evaluated using GraphPad Prism 6.0. Normalized data were statistically evaluated by one-column test, for multiple groups a Bonferroni multiplicity correction was introduced. Non-normalized groups were evaluated by students *t*-tests for comparison of two groups or by one-way or two-ANOVA followed by appropriate post-hoc tests for multiple comparisons, depending on the scientific question. Data with (adjusted) *p* values of <0.05 were considered statistically significant.

## Results

### Transcriptional profiling identifies AXL as a novel KLF2/KLF4 target in melanoma cells

**Fig. 1:**
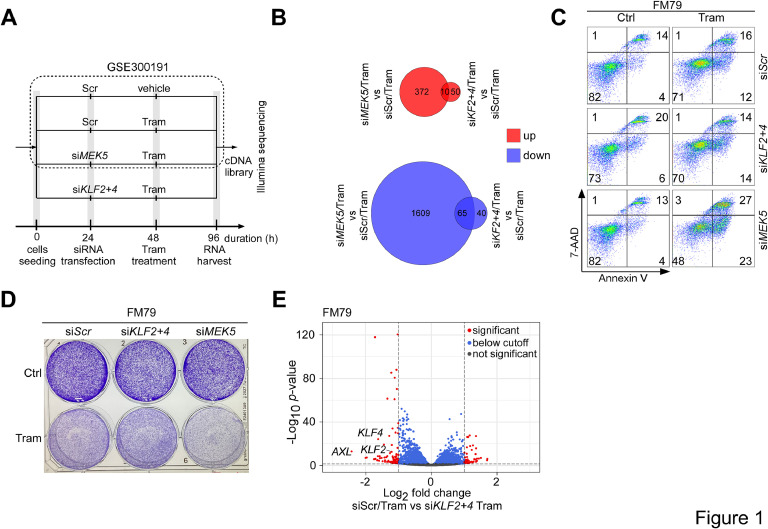
RNA Sequencing reveals AXL as KLF2/4 target.

The MEK5/ERK5 pathway has recently emerged as a major mechanism of therapy resistance, mediating the survival and proliferation of melanoma cells in response to targeted MEKi [4,7,13]. To gain insight into whether KLF2 and KLF4 might contribute to these processes or other aspects of MEKi resistance, such as phenotypic differentiation, immune suppression or invasion, we performed bulk RNA sequencing in FM79 cells. This *NRAS*-mutated melanoma cell line exhibits basal ERK5 activity, is highly sensitive to ERK5i/MEKi-induced apoptosis, and has previously been validated for ERK5-dependent KLF2/4 expression [4]. In these experiments, we compared FM79 cells exposed to Tram and transfected with a scrambled siRNA (siScr) with Tram-treated FM79 cells that were transfected with a pool of siRNAs targeting *KLF2* and *KLF4* (Fig. 1A, Supp. Fig. 1A, B). In addition, we compared the dataset with previously published sequencing data of si*MEK5*-transfected Tram-treated FM79 and untreated siScr-transfected FM79 cells [7] (GSE300191) that were generated in parallel. This yielded lists of differentially expressed genes (DEGs) (threshold: log2FC > 1, *p*.adj < 0.05), which we subsequently refer to as “Tram-regulated" (comparison siScr/control vs Tram), "si*KLF2/4*-regulated" (comparison siScr/Tram vs si*KLF2/4*/Tram) and “si*MEK5*-regulated" (comparison siScr/Tram vs si*MEK5*/Tram), respectively. Remarkably, only 165 genes were identified as si*KLF2/4*-regulated, while a total of 2056 genes were regulated by si*MEK5* (Fig. 1B). Only 75 DEGs were shared between both groups, indicating that only a small fraction was expressed in a KLF2/4-dependent manner. Unlike the si*MEK5*-regulated gene set [7], functional annotation cluster analysis using the publicly available Database for Annotation, Visualization, and Integrated Discovery (DAVID) [32] also did not reveal a pronounced enrichment of cell cycle- or apoptosis-regulating functional clusters among the si*KLF2/4*-downregulated DEGs. (Supp. Fig. 1C). Consistently, *KLF2/4* double knockdown did not sensitize FM79 cells to Tram-induced apoptosis while *MEK5* knockdown clearly augmented Tram-induced apoptosis in these cells (Fig. 1C, Supp. Fig. 2A). Unlike *MEK5* knockdown, siRNA-mediated *KLF2/4* depletion also did not enhance cell cycle arrest in Tram-treated FM79 (Fig. 1D) or BLM, a less apoptosis-prone *NRAS*-mutated melanoma cell line that lacks basal ERK5 activity but is highly susceptible for ERK5i/MEKi-induced cell cycle arrest [4,7] (Supp. Fig. 2B *top*). Expression controls excluded inefficient knockdown as explanation for these results (Supp. Fig. 2B *bottom*, data not shown). Together these data indicate that KLF2 and KLF4 are dispensable for the proliferative and cytoprotective effects of compensatory ERK5 activation in MEKi-exposed melanoma cells but control a more restricted set of genes that may regulate other aspects of MEKi resistance.

**A:** Schematic treatment scheme and timeline of siRNA transfection and Tram treatment of FM79 melanoma cells used for bulk RNA sequencing. Sequencing data from sample 1-3 have already been published elsewhere [7] (GSE300191).**B:** Venn diagrams, showing the number of DEGs regulated under MEKi by si*KLF2/4* versus si*MEK5*. Red circles indicate upregulated, blue circles >2 fold downregulated genes. si*MEK5*-regulated genes were part of an already published dataset [7] (GSE300191). **C:** Dot plots, showing representative (N=3) flow cytometric analyses of 72h medium- or 5 nM Tram-exposed FM79 melanoma cells after transfection with the indicated siRNAs and co-staining with the membrane impermeable DNA stain 7-amino-actinomycin D (7-AAD) and the apoptosis marker Annexin V as readout for cytotoxicity. Percentages of viable (7-AAD/Annexin V-negative) cells, early apoptotic (Annexin V single positive), late apoptotic (7-AAD/Annexin V double positive) and non-apoptotic dying cells (7-AAD single positive) are plotted in the lower left, lower right, upper right and upper left quadrants, respectively. A quantification of the mean percentage of viable cells from all three experiments is provided in Supp. Fig. 2A. **D:** Representative crystal-violet staining (n=4) of FM79 following transfection with siScr, si*KLF2+4* or si*MEK5* and reseeding at equal density into 6-well plates. Six hours after re-seeding cells were either treated with vehicle (Ctrl) or Tram and cells stained with crystal violet solution after 7 days, when control cells reached confluency. **E**: Volcano plot, depicting genes that were statistically up- (*right*) or downregulated (*left)* (cut-off: Log2 fold change >1, -Log10 adjusted *p*-value >1.3; dotted lines) in the comparison siScr/Tram versus si*KLF2+4*/Tram. Dark grey dots denote genes with non-significant change (Log2 fold change <1, -Log10 adjusted *p*-value <1.3), blue dots differentially expressed genes (DEGs) with Log2 fold change <1, and red dots si*KLF2/4*-regulated DEGs with Log2 fold change >1.

**Fig. 2:**
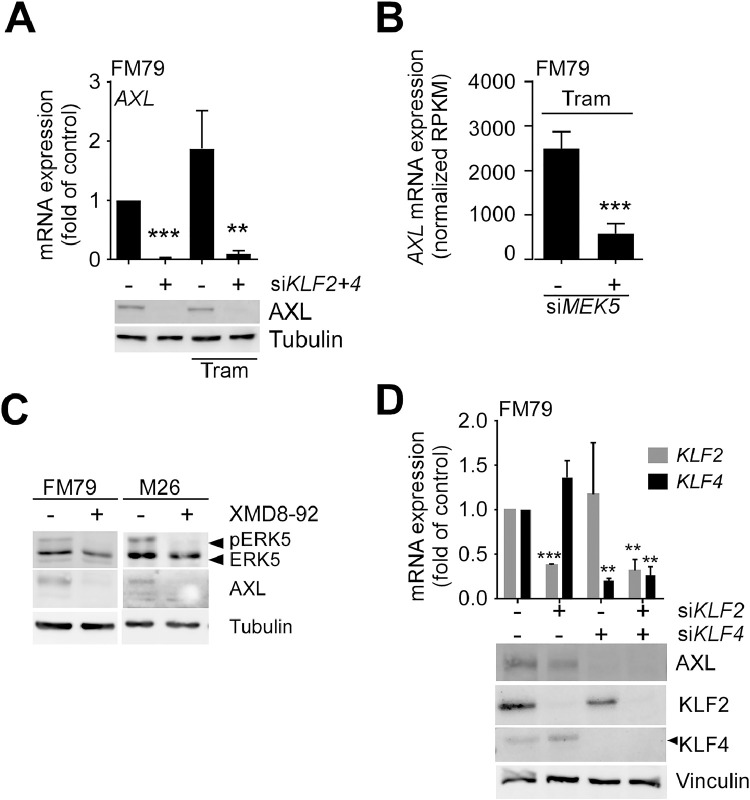
AXL expression is regulated by KLF4.

We thus specifically screened our list of DEGs (Supp. Table 5) for prominent genes that control other features of therapy resistance such as phenotypic switching. Remarkably, we identified AXL, a key receptor tyrosine kinase mediating tumor progression by promoting melanoma differentiation, invasiveness and metastasis [[33], [34], [35]], as top *KLF2/4* siRNA-suppressed gene in Tram-treated FM79 cells (Fig. 1E, Supp. Table 5). The dependency of AXL expression on KLF2/4 was subsequently confirmed by qPCR and immunoblot (Fig. 2A). In line with the previously observed dependence of KLF2/4 expression on ERK5 activity in FM79 cells [4], we were also able to validate *AXL* as *siMEK5*-regulated gene (Fig. 2B). In addition, treatment with the ERK5 inhibitor XMD8-92 [36] effectively suppressed basal AXL expression in both FM79 and M26 cells, a second *NRAS*-mutant melanoma cell line characterized by intrinsic ERK5 autophosphorylation [4] (Fig. 2C). Knockdown experiments with siRNAs specific for the individual KLFs subsequently revealed that basal AXL expression was only mildly affected by *KLF2* depletion but was completely abrogated by *KLF4* knockdown (Fig. 2D, Supp. Fig. 2C). In melanoma cells with endogenous ERK5 autophosphorylation basal AXL expression accordingly requires both ERK5 activity and KLF4 expression.

**A:** Effect of combined *KLF2* and *KLF4* knockdown (si*KLF2/4*) or siScr transfection (-) on *GAPDH*-normalized AXL mRNA expression (bar diagram, mean +SD, N=3) (*top*) or protein levels (representative immunoblot of N=3, *bottom*) in FM79 treated with or without 5 nM Tram for 48h, respectively. **B:** Bar diagram, showing *AXL* mRNA levels in response to the indicated treatments expressed as average normalized Reads Per Kilobase Million (RPKM) +SD of N=3 independent experiments. Data were extracted from a previously published dataset (GSE300191) [7]. **C:** Immunoblots for AXL and ERK5 with total lysates of FM79 and M26 cells treated with either vehicle or 5 µM of the ERK5i XMD8-92 for 48h. **D***top***:** Bar diagram showing the *GAPDH*-normalized *KLF2* and *KLF4* mRNA levels after transfection with pools of each two individual siRNAs against *KLF2, KLF4* or both *KLFs* (mean +SD, N=3)*. bottom*: Corresponding immunoblots representative of N=4 independent experiments, confirming efficiencies of single or combined *KLF2* and *KLF4* knockdown at protein level and their effects on AXL protein expression. Densitometric quantification of mean loading control-normalized AXL protein suppression + SD in the four performed siRNA experiments is provided in Supp. Fig. 2C. Tubulin or Vinculin blots in (**A, C** and **D)** served as loading control. Statistical differences in relation to the siScr control group (-) in the bar diagrams was evaluated by one-column test with (**A, D**) or without (**B**) Bonferroni correction. *: *p*<0.05, **: *p*<0.01, ***: *p*<0.001.

### MEKi-induced AXL expression in melanoma cells is mediated by the ERK5/KLF4 axis

**Fig. 3:**
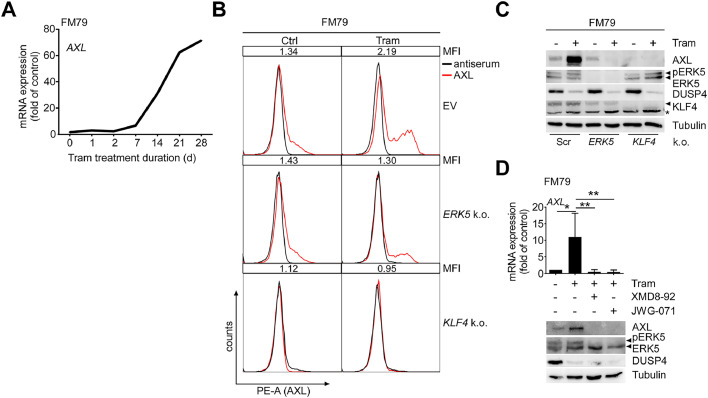
ERK5i represses MEKi-induced AXL induction.

In multiple cancer entities, AXL upregulation is closely associated with therapy resistance [[37], [38], [39]]. In melanoma, BRAFi/MEKi resistance correlates with AXL expression [40,41]. As drug-naïve FM79 show only a low percentage of AXL positivity and consequently express relatively low total AXL protein concentrations compared to other melanoma cell lines [7], we tested whether AXL expression might further increase during development of MEKi resistance. Indeed, treatment of FM79 cells with 5 nM Tram for a period of 1 to 28 days resulted in a steep rise in *AXL* mRNA expression starting about 14 days after treatment (Fig. 3A). At protein level this coincided with increased surface and total protein expression (Supp. Fig. 3, Fig. 3B, C). Intriguingly, both CRISPR/*Cas9*-mediated disruption of *ERK5* or pan*KLF4* and co-treatment with the ERK5i XMD8-92 or the newer, more specific compound JWG-071 [42] could prevent the increase of AXL protein in response to prolonged Tram-treatment in FM79 (Fig. 3B,-D). Consequently, MEKi resistance-associated AXL induction also depends on ERK5 and KLF4.

**A:** Line diagram, depicting gradual induction of AXL mRNA expression upon 5 nM Tram exposure over time in days (d). Shown is a representative experiment out of N=4 with data expressed as *GAPDH*-normalized fold mRNA expression of control (0d Tram). **B, C:** Representative (N=3) histograms overlays of flow cytometric surface staining for AXL (red) and the respective control antiserum staining (black) (**B**) or AXL immunoblots with total lysates (**C**) of either empty vector (EV), stably scrambled (Scr, **C**), *ERK5* or *KLF4* gRNA expressing (k.o.) FM79 cells treated with vehicle or 5 nM Tram for a period of two weeks. Quantifications of (**B**) are presented in Supp. Fig. 3. **D:** Bar diagram illustrating mean *GAPDH*-normalized *AXL* mRNA expression +SD (N=3) (*top*) and a corresponding representative immunoblot of N=3, showing AXL protein levels in total lysates (*bottom*) of FM79 treated for two weeks with Tram in presence or absence 5 µM of the indicated ERK5i. As knockdown control in (**C**) an immunoblot for KLF4 from the same experiment is shown with the band corresponding to KLF4 highlighted by an arrow. *Indicates an unspecific band frequently observed with the employed anti-KLF4 antiserum. Additional immunoblots for ERK5, the ERK1/2 target DUSP4, and Tubulin in (**C**) and (**D**) served as functional controls for successful ERK5 gene disruption/inhibition, Tram-mediated MEK1/2 inhibition and equal loading, respectively. Statistical significance was evaluated by one-column test followed by Bonferroni correction (comparison between control and the Tram-treated group) or one-way ANOVA post Dunnett’s test (comparison between the Tram- and the Tram/ERK5i-treated groups), respectively (*: *p*<0.05, **: *p*<0.01).

**Fig. 4:**
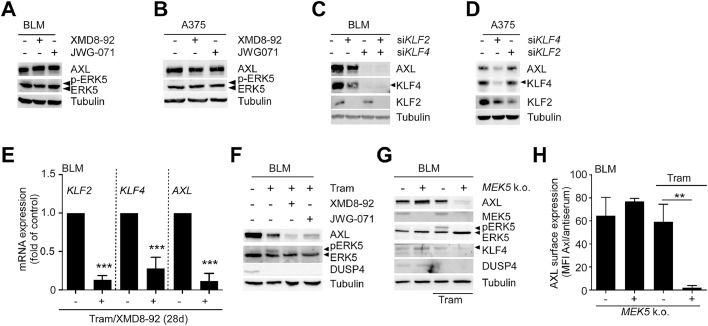
Sustained MEKi renders basal AXL expression in ERK5 phosphorylation-negative metastatic melanoma cell lines susceptible to ERK5i.

In our previous study, we noticed that some strongly metastatic melanoma cell lines such as the *NRAS*-mutant BLM cell line express high steady state levels of AXL protein but lack ERK5 basal activity [7]. This suggests that AXL expression is also governed by ERK5-independent pathways. To explore this, we treated drug-naïve BLM cells with the ERK5 inhibitors XMD8-92 or JWG-071. Fig. 4A and B illustrate that neither treatment with XMD8-92 nor JWG-071 affected AXL protein level in either BLM or in the *BRAF*-mutant melanoma cell line A375, which similarly shows high levels of basal AXL protein expression but no endogenous ERK5 phosphorylation. However, siRNA-mediated *KLF4* depletion, was still capable of suppressing AXL levels in these metastatic cell lines while *KLF2* knockdown had only mild effects (Fig. 4C, D, Supp. Fig. 4). A similar requirement of KLF4 but not of KLF2 was also observed in the ERK5-phosphorylation negative *NRAS-*mutant cell line MaMel26 that also expresses high basal AXL levels [7] and the BRAF mutant cell line FM88 (Supp. Fig. 4B, C). Remarkably, sustained MEKi/ERK5i co-inhibition of BLM for two to four weeks not only inhibited MEKi-induced ERK5 phosphorylation and *KLF2* and *KLF4* transcription but also markedly reduced AXL expression at the mRNA and protein level (Fig. 4E, F). Similarly, CRISPR/*Cas9*-mediated *MEK5* disruption alone in BLM did not affect AXL basal protein but efficiently suppressed AXL total and surface protein expression, KLF4 levels, and MEKi-induced ERK5 phosphorylation under conditions of sustained Tram exposure (Fig. 4G, H). Prolonged treatment conditions promoting ERK5-dependent MEKi resistance therefore may render AXL expression susceptible for ERK5 inhibition, even in cell lines where high basal AXL expression is driven by ERK5-independent mechanisms.

**A, B:** Representative immunoblots of N=3 experiments, showing AXL protein expression in response to treatment of BLM (**A**) or A375 (**B**) cells with the indicated ERK5i for 48h. An ERK5 blot using an antiserum detecting both unphosphorylated ERK5 and its autophosphorylated, slower migrating form is shown to confirm lack of ERK5 activity in the respective drug-naïve and ERK5i-treated cells. Tubulin blots served as loading control. **C, D:** Representative immunoblots of N=3 with total lysates of BLM (**C,** for corresponding densitometric quantification of all three experiments compare Supp. Fig. 4A) or A375 cells (**D**) harvested 48h after transfection with scrambled siRNA (-) or with pools of each two different siRNAs directed against *KLF2, KLF4* of both *KLFs*. Additional immunoblots for KLF4, KLF2 and Tubulin served as knockdown or loading control, respectively. **E:** Bar diagram, showing mean (N=3) fold *GAPDH*-normalized mRNA expression +SD of the indicated genes in BLM cells co-treated for 28 days with Tram/XMD8-92. **F, G:** Representative immunoblots of AXL expression in wildtype BLM treated for two weeks as indicated (**F**), or *MEK5* knockout (k.o.) BLM versus empty vector-transduced BLM (-) treated with Tram- or vehicle for 14 days. Immunoblots for ERK5 are shown as validation of Tram-induced ERK5 phosphorylation or lack thereof in response to ERK5i or *MEK5* disruption, respectively. Immunoblots for Tubulin and the ERK1/2 target DUSP4 blot served as loading control and Tram functionality control, respectively. In (**G**) additionally a MEK5 immunoblot is included as knockout control. **H:** Flow cytometric quantification of mean (N=3) median fluorescence intensity (MFI) ratios of AXL surface staining and its corresponding control antiserum staining +SD of *MEK5* k.o. BLM or a respective empty-vector cell line (-) treated with Tram or vehicle for 14 days. Statistical difference to the respective untreated control in (**E**) was evaluated by one-column test. Statistical difference between the indicated groups in (**H**), was tested unpaired students *t*-test. (**: *p*<0.01). Tram was used at 25 nM and the respective ERK5i at 5 µM in all experiments.

### MEKi/ERK5i is sufficient to counteract MEKi-induced AXL expression in melanoma

**Fig. 5:**
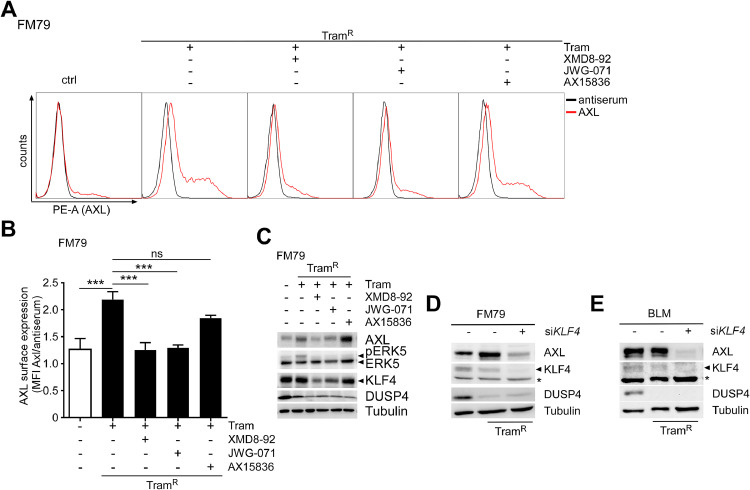
ERK5i triggers rapid loss of AXL expression in Tram^R^ cells.

We have previously shown that ERK5i addition can rapidly re-sensitize MEKi-resistant melanoma cells for MEKi-induced cell cycle arrest [7]. In order to explore if ERK5i co-treatment can also antagonize MEKi resistance-associated AXL expression, we treated FM79 cells with Tram for two weeks to induce Tram resistance (Tram^R^). Subsequently, we re-seeded the cells at equal density into Tram-containing medium and co-incubated them with different ERK5i for 48h. Interestingly, compounds that inhibit both ERK5 kinase activity and its transactivation function such as XMD8-92 and JWG-071 significantly suppressed KLF4 and AXL total protein expression as well as AXL surface protein in Tram^R^ FM79 cells. By contrast, AX15836 that solely acts as kinase inhibitor [43] only weakly affected AXL and KLF4 expression (Fig. 5A–C). Consistent with a role of KLF4, transfection of *KLF4* siRNA into Tram^R^ FM79 or BLM was also capable of suppressing AXL expression (Fig. 5D, E). Hence, the increased AXL expression in MEKi-resistant cells is not merely a consequence of compensatory ERK5 kinase activity but also requires the transactivation function of ERK5 that steadily fuels KLF4 mRNA supply.

FM79 cells were pre-treated with 5 nM Tram for two weeks and re-seeded into Tram-containing medium. The following day, cells were co-treated with diluent or 5 µM of the indicated ERK5i for 48h **A:** Representative histograms overlays of N=3 flow cytometric stainings with a control antiserum (black) or an antiserum against AXL (red) for surface expression. **B:** Bar diagram, showing mean ratios of median fluorescence intensity (MFI) of AXL surface staining and control antiserum staining +SD of N=3 independent experiments performed as in (**A**). Statistical significance was evaluated by one-way ANOVA post Dunnett’s test, comparing all samples to the Tram mono-treated group. NS: p>0.05, ***: *p*<0.001. **C:** Immunoblots representative of N=3 experiments performed in parallel to **A. D, E:** Representative immunoblots of N=3 experiments illustrating AXL expression in Tram^R^ FM79 cells (**D**) or Tram^R^ BLM (**E**) 48h after transfection with a scrambled siRNA (-) or an siRNA against pan*KLF4*. * Indicates an unspecific band. An ERK5 blot in (**C**) served to control for efficient kinase inhibition by the respective ERK5i. An immunoblot of the ERK5 transcriptional target KLF4 was included as readout for suppression of the transactivation function by the employed ERK5i **(C)** and to confirm efficient knockdown by *KLF4* siRNA transfection in (**D** and **E,** arrow: KLF4 band, *: unspecific band). DUSP4 and Tubulin blots were included as functionality control for Tram or loading control, respectively. Employed Tram concentrations were 5 nM (FM79) or 25 nM (BLM); XMD8-92, JWG-071 and AX15836 were used at 5 µM in all experiments.

### MEK5/ERK5/KLF4 signaling drives invasion/migration through AXL regulation

**Fig. 6:**
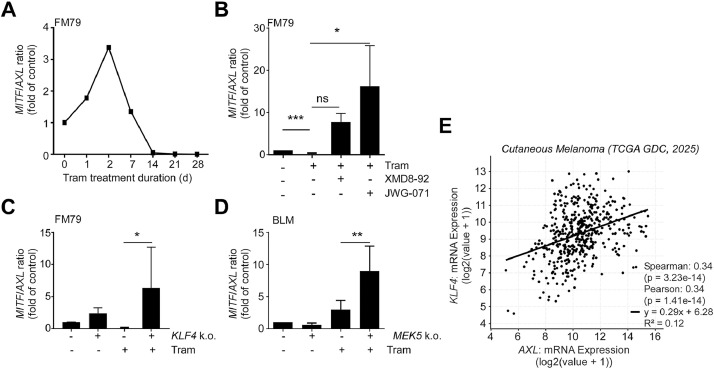
ERK5 /KLF4 inhibition increases the MITF/AXL ratio of MEKi-resistant melanoma.

**Fig. 7:**
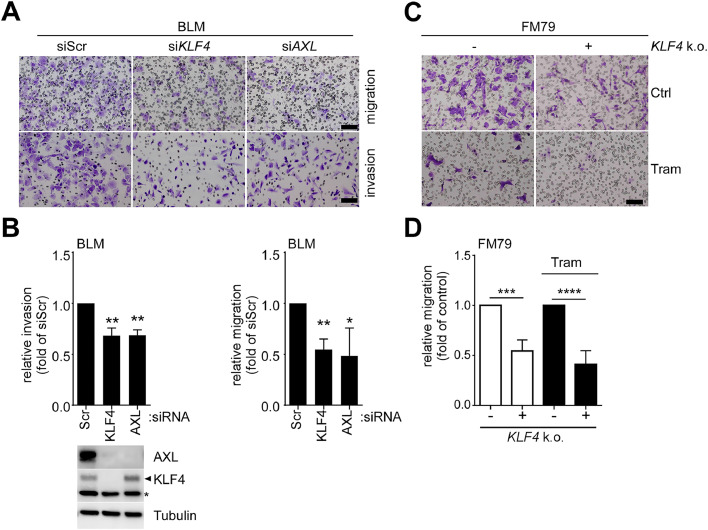
KLF4-depleted cells show decreased migration and invasion capacity.

A low *MITF/AXL* ratio is widely recognized as a reliable predictor of targeted MAPKi resistance in melanoma [40]. Moreover, high AXL expression has been implicated in promoting the migration and invasion capacity of different tumor cells including melanoma both *in vitro* and *in vivo* [44,45]. To examine the *MITF/AXL* ratio in our settings, we quantified *MITF* and *AXL* mRNA expression by qRT-PCR in different pharmacological and genetic setups. Following prolonged MEKi in FM79, the *MITF/AXL* ratio initially increased over the first two days, but subsequently declined and flattened after two weeks, a pattern indicative of acquired resistance (Fig. 6A). However, co-inhibition with MEKi and ERK5i dramatically increased the *MITF/AXL* ratio in FM79 cells (Fig. 6B). A similar trend was observed in *KLF4* knockout FM79 cells (Fig. 6C) and *MEK5*-deficient BLM (Fig. 6D). Moreover, a significant positive correlation was observed between *AXL* and *KLF4* mRNA expression in cutaneous melanoma patients (https://www.cancer.gov/tcga, Fig. 6E). These data indicate that the MEK5/ERK5/KLF4 axis may critically influence invasiveness and the metastatic potential of melanoma cells. Indeed, *AXL* and *KLF4* knockdown resulted in a similar suppression of migration and invasion of BLM cells in Transwell assays (Fig. 7A, B). Likewise, *KLF4*-deficient FM79 showed a markedly decreased migration capacity compared to control cells, under both basal conditions and MEKi treatment (Fig. 7C, D). Together, these data imply KLF4 as a novel regulator of the metastatic potential and invasiveness of *NRAS*-mutant melanoma cells by controlling AXL expression.

**A:** Line diagram representative of N=4 experiments, depicting gradual changes in the *MITF*/*AXL* mRNA expression ratio in response to sustained Tram exposure over time in days (d). Data are presented as *GAPDH*-normalized fold ratio of *MITF* and *AXL* mRNA expression in relation to the untreated control (0d Tram). **B:** Changes in the *MITF*/*AXL* mRNA expression ratio in FM79 cells treated with indicated inhibitors for two weeks. **C, D:***MITF*/*AXL* mRNA expression ratio in *KLF4* knockout FM79 cells (**C**) or *MEK5*-knockout BLM (k.o.) (**D**) and their corresponding empty vector transduced cell lines (-) after treatment with vehicle or Tram for two weeks. Bar diagrams in (**B**-**D**) each represent mean fold ratios of *GAPDH*-normalized *MITF* and *AXL* mRNA expression +SD relative to the respective drug-naïve control cells and were obtained from N=3 independent experiments. For **B-D**, Statistical significance to the indicated group was evaluated by one-column test followed by Bonferroni correction (**B,** Tram monotreatment set to 1) or two-way ANOVA post Sidak’s test (**C-D**). ns: p>0.05, *: *p*<0.05, **: *p*<0.01***: *p*<0.001. **E:** Scatter plot depicting the mRNA expression levels (FPKM z-scores) of *AXL* and *KLF4* in cutaneous melanoma patients. Data were retrieved from cBioPortal (Cutaneous Melanoma TCGA GDC 2025). Spearman’s and Pearson’s rank correlation coefficients are shown with their associated *p*-values. The solid line represents the linear regression, and R^2^ indicates the coefficient of determination.

(**A, C**): Representative results of Transwell-based migration and invasion assays with BLM cells transfected with the indicated siRNAs (**A**), or invasion assays of treatment-naïve or 14 days Tram-treated FM79 stably expressing a scrambled gRNA (-) or a gRNA against pan*KLF4* (**C**). Cells were starved in 0.5 % FCS-containing media for 24h prior to reseeding onto the top layer of Trans-well migration or invasion chambers and exposure to 10% FCS-containing medium for 24h. Membranes with migrated or invaded cells were then fixed, stained with crystal-violet and photographed. Scale bar: 100 µM. (**B, D):** Quantification of N=4 experiments performed as in (**A**) and (**C**). For each experiment five representative visual fields were counted and data related to the respective experimental controls (set to 1). Bar diagrams represent mean fold migration (**B**, *right,***D**) or invasion (**B**, *top left*) +SD, respectively. (**B***bottom left*): Immunoblots, confirming KLF4 and AXL knockdown for the experiment shown in **A**. A Tubulin blot served as a loading control. * Indicates an unspecific band. Statistical significance in relation to the respective experimental control (set to 1) was evaluated by one-column test with (**B**) or without (**D**) Bonferroni correction. *: *p*<0.05, **: *p*<0.01.

## Discussion

The two Krüppel-like factors KLF2 and KLF4 are key effectors of the MEK5/ERK5 pathway in endothelial cells, where they mediate the bulk of transcriptional responses to ERK5 activation [21]. However, KLF2/4 expression has been found to correlate with ERK5 activation also in other cell types, including *NRAS*-mutant melanoma [4,[23], [24], [25], [26], [27]]. In this study, we analyzed the role of KLF2 and KLF4 in the context of MEKi-resistance in *NRAS*-mutant melanoma in which ERK5 activation enables tumor cells to remain proliferative and survive the therapeutic intervention [4].

Intriguingly, both our *in vitro* assays and our bulk RNA sequencings indicate that KLF2 and KLF4 are not relevant for the proliferative and anti-apoptotic effects of MEKi-induced ERK5 activation in *NRAS*-mutant melanoma cells. This was unexpected since it was previously shown that at least KLF4 can control proliferation or apoptosis resistance in various cell types. For instance, both forced KLF4 expression and constitutive ERK5 activation were able to protect endothelial cells from growth factor deprivation-induced apoptosis [21]. KLF4 has also been identified as context-specific oncogene capable of suppressing senescence induction by oncogenic RAS [19] and has previously been implicated in the regulation of proliferation and survival of *BRAF*-mutant melanoma cells [46], a finding that we could not reproduce for *NRAS*-mutant melanoma. Rather we observed a slightly increased survival/proliferation upon combined KLF2/4 depletion of our Tram-treated cell lines, which is more in line with a role of KLF2 and KLF4 as classical tumor suppressors [19,47,48] and supports the concept that the proliferative function of KLF4 is highly context-specific [19]. It should also be noted in this respect that we used pan-specific si- and gRNAs in order to target all known KLF4 isoforms including KLF4α, a recently described KLF4 splice variant with putative oncogenic function [30,31], ruling out that our results are caused by depletion of a single-isoform.

A second surprise was that in *NRAS*-mutant melanoma cells, unlike endothelial cells, only a small fraction of the ERK5-dependent transcriptional response was KLF2/4-dependent. Remarkably, however, we identified the pro-metastatic receptor tyrosine kinase AXL as a common target of ERK5 and KLF4. Of note, this was only fully true under conditions of experimentally induced MEKi resistance that promotes AXL expression as a result of phenotypic switching of melanoma cells to a highly invasive, aggressive phenotype [40,41]. By contrast, the high basal AXL expression in the metastatic melanoma cell lines BLM and A375, which both exhibit no basal ERK5 autophosphorylation activity, was not dependent on ERK5. Nonetheless, AXL expression was still sensitive to *KLF4* depletion in these cells and could be blocked by prolonged MEKi/ERK5i co-treatment. This indicates that in these metastatic cell lines the endogenous expression of KLF4 and AXL is independent of ERK5 but becomes sensitive to ERK5i under therapeutic MAPKi. Intriguingly, it was previously reported that also ERK1/2 can regulate KLF4 expression in several melanoma cell lines including A375 [46]. Therefore, it is conceivable that, under constant MEKi-induced therapeutic stress, cells with high intrinsic AXL/KLF4 expression switch from default ERK1/2 signaling to ERK5 signaling to sustain KLF4 and AXL expression, making them susceptible to ERK5i. Indeed, our observation that AXL expression remained unchanged in untreated *MEK5*-deficient BLM but was lost under prolonged Tram-treatment would support this hypothesis.

In several tumors increased AXL expression has been linked to a pro-migratory, metastatic phenotype [49]. In agreement, we found that both *AXL* and *KLF4* depletion/disruption decreased the migratory/invasive potential of our *NRAS*-mutant melanoma cell lines. Moreover, we observed that the MITF/AXL ratio in Tram-exposed cells increased strongly both upon pharmacological ERK5i or genetic disruption of *MEK5* or pan*KLF4*. While the exact functional consequence of the observed switching to an AXL-low phenotype requires further investigation in appropriate *in vivo* models, AXL has shown to be a promising therapeutic target for both BRAFi/MEKi-resistant melanoma and immunotherapy-resistant melanoma in pre-clinical studies [41,50]. Consequently, interference with ERK5/KLF4 signaling may not only be an attractive strategy to prevent resistance to MAPKi-based targeted therapy but also might be a promising approach to limit resistance rates of current immunotherapies.

## Ethics approval

No ethical considerations were required for the conducted study.

## Data availability statement

RNA-seq data are deposited in the GEO database (accession GSE319305). Materials and data generated during the study will be provided by the corresponding author upon reasonable request.

## CRediT authorship contribution statement

**Rupesh Paudel:** Writing – original draft, Visualization, Validation, Investigation, Formal analysis, Conceptualization. **Simon Goller:** Visualization, Validation, Investigation, Formal analysis. **Stefanie Schwarz:** Validation, Investigation, Formal analysis. **Katharina Meder:** Validation, Investigation, Formal analysis. **Matthias Goebeler:** Writing – review & editing, Resources. **Marc Schmidt:** Writing – review & editing, Writing – original draft, Visualization, Validation, Supervision, Resources, Project administration, Methodology, Investigation, Funding acquisition, Formal analysis, Data curation, Conceptualization.

## Declaration of competing interest

The authors declare that they have no known competing financial interests or personal relationships that could have appeared to influence the work reported in this paper.

## Appendix Supplementary materials

Image, application 1
