# Supplementary material for: AXL is a novel ERK5/KLF4 target in MEK inhibitor-treated melanoma
Source: Neoplasia. 2026 Mar 30;76:101301. doi: 10.1016/j.neo.2026.101301 (PMC13068529; doi:10.1016/j.neo.2026.101301)
Supplement: Supplementary file 1 [file mmc1.pdf]

SUPPLEMENTAL INFORMATION

to

AXL is a novel ERK5/KLF4 target in MEK inhibitor-treated melanoma

Rupesh Paudel[a], Simon Goller[a], Stefanie Schwarz[a], Katharina Meder[a], Matthias Goebeler[a], Marc Schmidt[a#]

[a]Department of Dermatology, Venereology and Allergology, University Hospital Würzburg, Josef-Schneider-Str. 2, 97080 Würzburg, Germany.

[#]Correspondence to: Prof. Dr. Marc Schmidt, Department of Dermatology, Venereology and Allergology, University Hospital Würzburg, Josef-Schneider-Str. 2, 97080 Würzburg, Germany. E-mail: schmidt_M11@ukw.de, Phone: +49-931-201-26396

This file contains:

Legends for

Supp. Figures

Supp. Tables

Supp. References

Supplementary Figure Legends

Supp. Figure 1: Functional annotation cluster analysis of the KLF2/KLF4-downregulated genes reveals no major alteration in apoptotic and proliferative gene sets

A, B: mRNA expression of *KLF2* (**A**) or *KLF4* (**B**) presented as mean normalized Reads Per Kilobase Million (RPKM) +SD, Data were extracted from N=3 RNA-seq experiments performed with FM79 treated as indicated. **C**: Top ten functional annotation clusters identified for the si*KLF2/4* downregulated genes. Tram was used at 5 nM in all experiments. Statistical significance was evaluated by one-way ANOVA post Dunnett's test. Compared groups are indicated. ***: $p<0.001$.

Supp. Figure 2: KLF2/KLF4 depletion does not augment MEKi-induced cytotoxicity and cell cycle arrest in melanoma

A: Flow cytometric quantification of mean cell viability +SD as determined by N=3 7-AAD and annexin V stainings of FM79 melanoma cells transfected with si-scrambled (siScr), si*KLF2+4* or si*MEK5* and treated with or without 5 nM trametinib (Tram) (Ctrl) for 72h. **B** *top*: Representative

crystal-violet staining (N=4) of BLM following transfection with siScr, si*KLF2+4* or si*MEK5* and reseeding at equal density into 6-well plates. Six hours after re-seeding cells were either treated with vehicle (Ctrl) or Tram and cells stained with crystal violet solution after 5 days, when control cells reached confluency. *bottom***:** Corresponding knockdown control for the crystal-violet assays above. Bar diagrams represent *GAPDH*-normalized mean (N=4) mRNA expression +SD of the indicated genes with statistical significance evaluated one-column *t*-test. **: $p<0.01$. **C**: Densitometric quantification of the mean loading control-normalized AXL protein expression + SD in immunoblots of N=4 independent knockdown experiments performed with FM79 analogous to Figure 2D. Data show ratios of the determined background-corrected grey values of the AXL band to the corresponding loading control band, normalized to the siScr-transfected experimental control (-) arbitrarily set to 1. Statistical differences between the specified siRNA-transfected samples and the siScr-transfected control are indicated (*$p<0.05$, ***$p<0.001$, one-column *t*-test with Bonferroni multiplicity correction).

Supp. Figure 3: KLF4 disruption prevents MEKi-induced AXL expression

Flow cytometric quantification of AXL surface expression in FM79 cells stably expressing empty vector (EV), ERK5 or panKLF4 gRNA (k.o.) and treated with or without 5nM Tram for 14 days. The bar diagram shows mean median fluorescence intensity ratios of N= 3 AXL surface stainings and their control antiserum-stained counterparts +SD, with data normalized to the unstimulated EV control (arbitrarily set to 1). Statistical differences between the Tram-stimulated empty vector control and the specified Tram-treated k.o. cell lines are indicated by asterisks (*** $p<0.001$, two-way ANOVA, Sidaks test)

Supp. Figure 4: KLF4 sustains the high basal AXL expression in ERK5 phosphorylation negative melanoma cell lines

A: Bar diagram, showing the mean loading control-normalized AXL expression +SD in immunoblots of N=3 knockdown experiments performed with the metastatic ERK5 phosphorylation-negative BLM cell line analogous to Fig. 4C. Data show ratios of the determined background-corrected grey values of the respective AXL band to the corresponding tubulin loading control band, normalized to the siScr-transfected experimental control (-) arbitrarily set to 1. Statistical differences between the specified siRNA-transfected samples and the siScr-transfected control are indicated (*$p<0.05$, ***$p<0.001$, one-column *t*-test with Bonferroni multiplicity correction). **B**, **C**: AXL immunoblots performed with total cell lysates from *NRAS*-mutant MaMel26a (**B**) or *BRAF*-mutant FM88 melanoma cells (**C**) harvested 48h after transfection with scrambled

siRNA (-) or with pools of each two different siRNAs directed against KLF2, KLF4. Additional immunoblots for KLF4, KLF2 and Tubulin served as knockdown or loading control, respectively.

Supp. Table 1: List of guide RNA sequences used in the manuscript

Supp. Table 2: List of siRNA sequences used in the manuscript

Supp. Table 3: List of RTqPCR primers used in the manuscript

Supp. Table 4: List of primary antibodies used in the manuscript

Supp. Table 5: List of statistically significant (≥2.0-fold) siKLF2/KLF4- downregulated genes

Supp. References

Paudel, R., Goller, S., Deutzmann, F., Gillitzer, A., Meder, K., Knorz, A., Schrama, D., Goebeler, M., & Schmidt, M. (2025). MEK5/ERK5 inhibition sensitizes NRAS-mutant melanoma to MAPK-targeted therapy by preventing Cyclin D/CDK4-mediated G1/S progression. *Cell Death Dis*, *16*(1), 689. https://doi.org/10.1038/s41419-025-08036-7

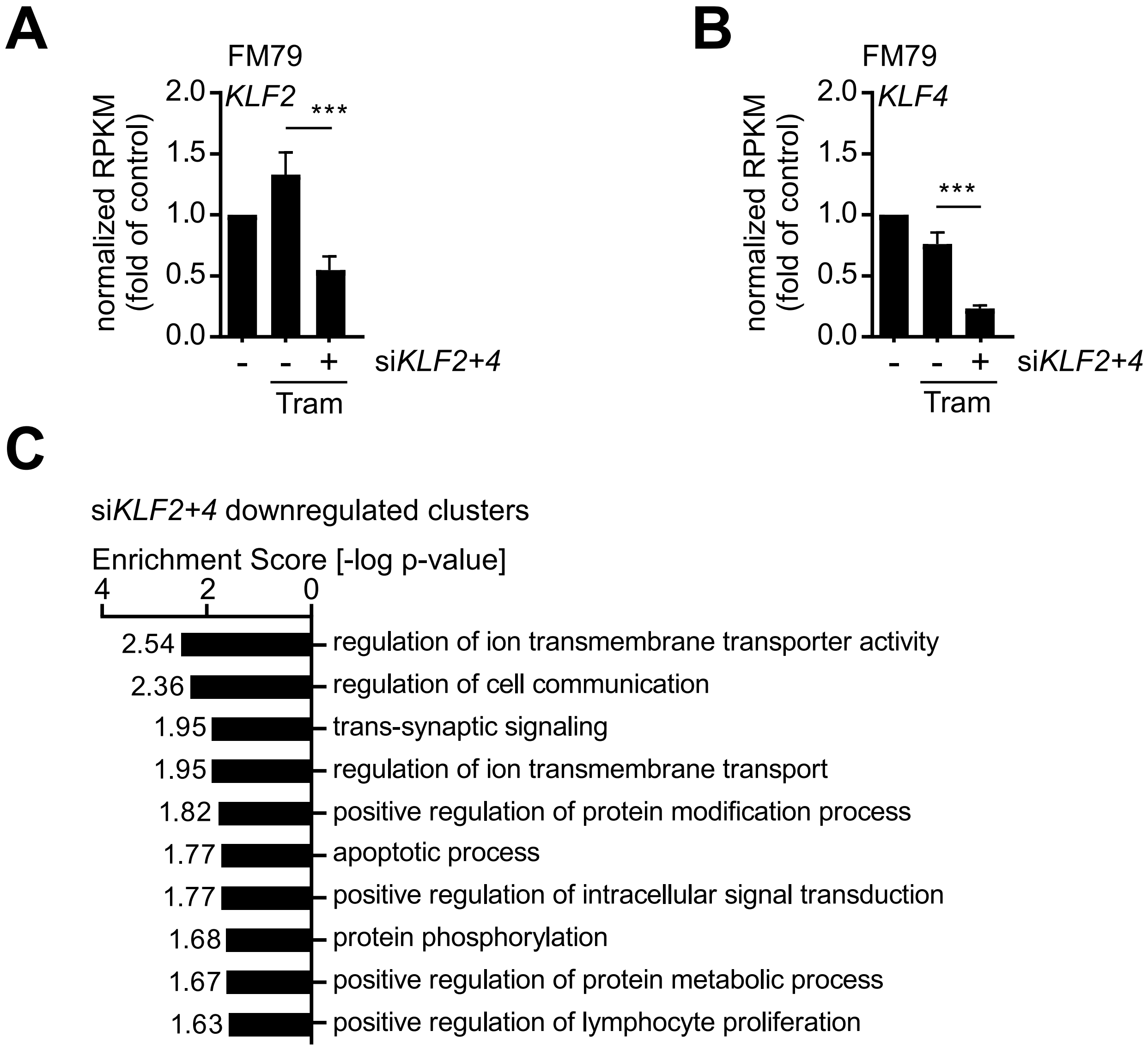

Supp. Figure 1

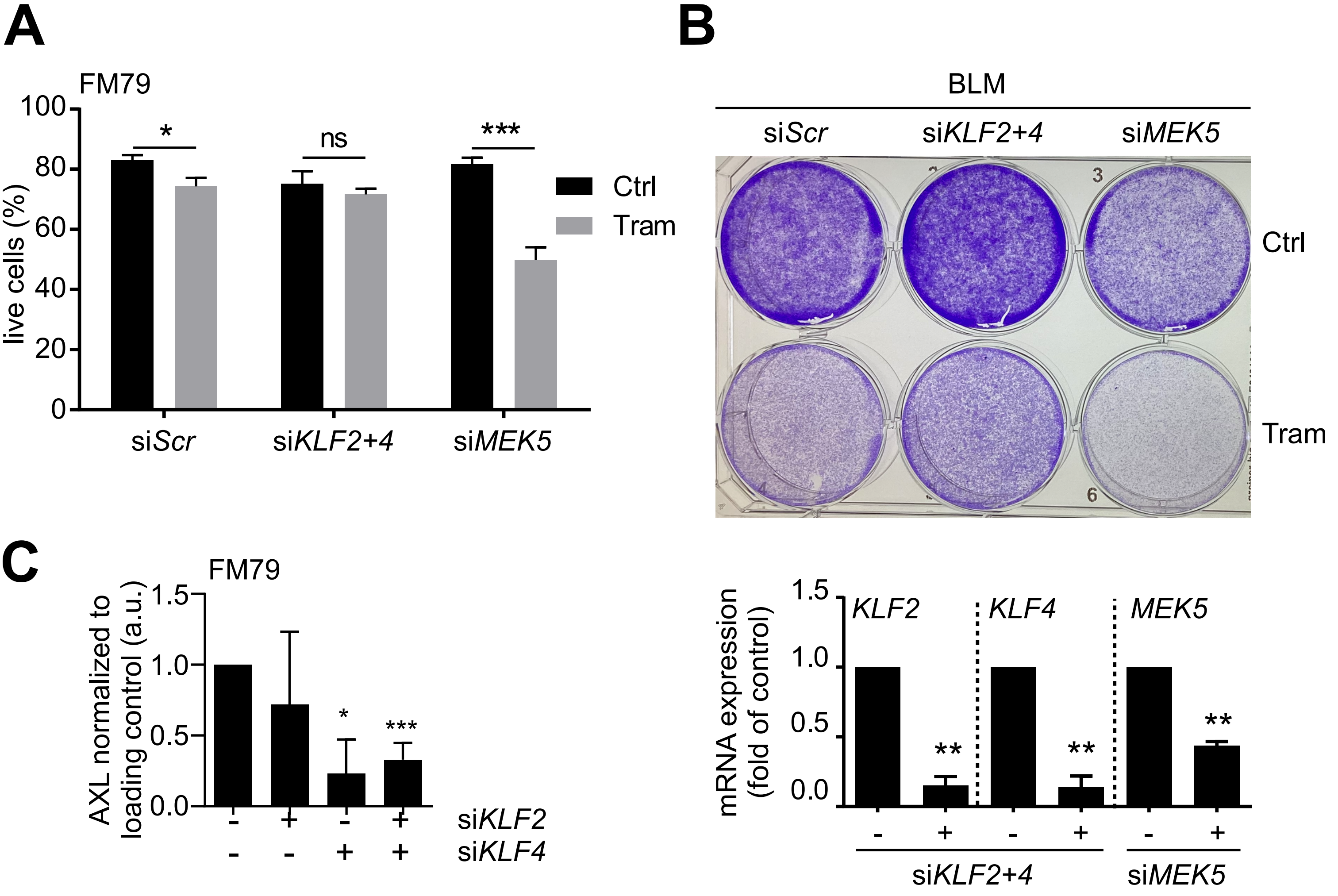

Supp. Figure 2

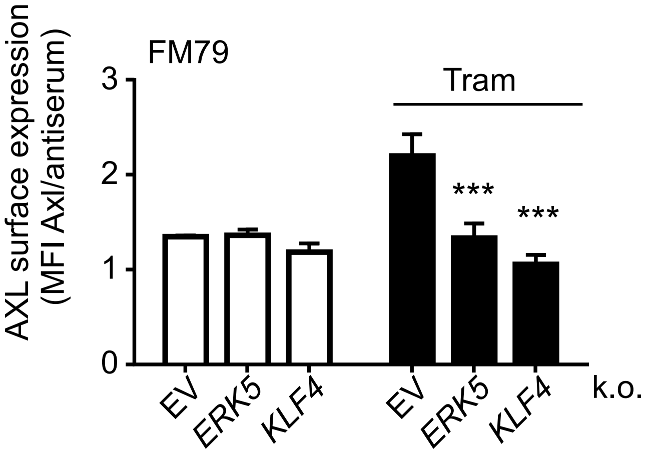

Supp. Figure 3

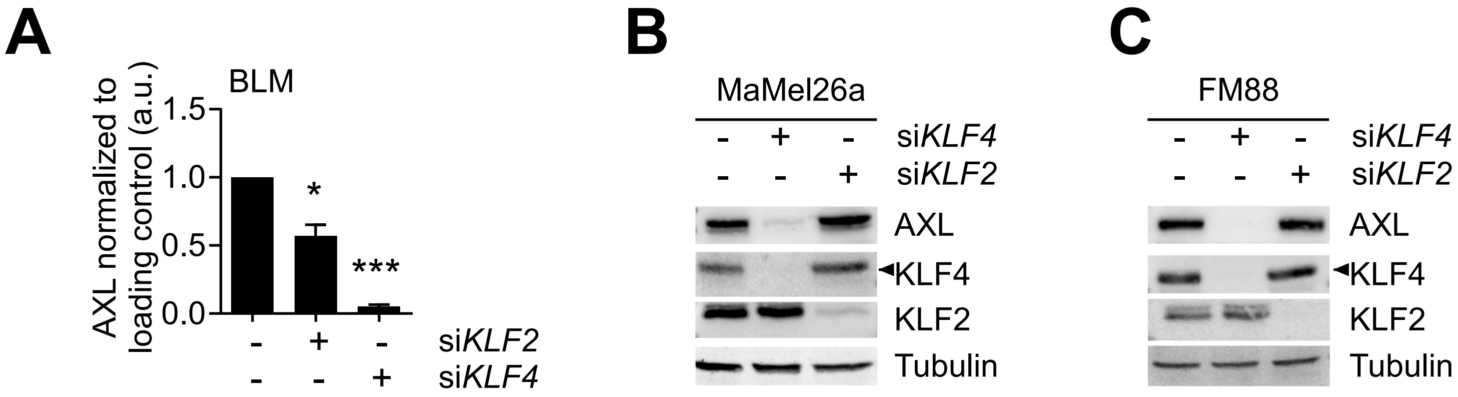

Supp. Figure 4

Supp. Table 1: List of gRNA sequences

Serial Number	siRNA	gRNA sequence	Source
1	Scrambled_A	CCACACCTGTCTAGCATGAC	Human CRISPR Knockout Pooled library
2	MAPK7_B	TGACCGCGAAGCCCTCACTC	Human CRISPR Knockout Pooled library
3	KLF4_F	AGCGATACTCACGTTATTCG	Self designed using CHOPCHOP

Supp. Table 2: List of RTqPCR primers

serial number	Gene	Forward primer (5'-3')	Reverse primer (5'-3')
1	*GAPDH*	CCACCCATGGCAAATTCC	GATGGGATTTCCATTGATGACA
2	*KLF2*	CTACACCAAGAGTTCGCATCTG	CCGTGTGCTTTCGGTAGTG
3	*KLF4*	ACCAGGCACTACCGTAAACACA	GGTCCGACCTGGAAAATGCT
4	*MEK5*	CCGGACCCTCTCAACACAG	ATGACCAAGAGTGTCCCGATA
5	*AXL*	ATCAGCTTCGGCTAGGCAG	TCCGCGTAGCACTAATGTTCT
6	*MITF*	TCACAACCTGATTGAACGAAGAA	ACTTTCGGATATAGTCCACGGAT

Supp. Table 3: List of siRNA sequences

Serial Number	siRNA	siRNA Target sequence	Product identifier	Supplier
1	siScrambled	AATTCTCCGAACGTGTCACGT	#1022563	Qiagen, Hilden, Germany
2	siKLF2_1	TTGTATATAGTGACTGACAAA	#SI03246096	Qiagen, Hilden, Germany
3	siKLF2_2	ACACCAAGAGTTCGCATCTGA	#s20270	Ambion, Waltham, Massachusetts USA
4	siKLF4_1	TTGGTGAGTCTTGGTTCTAAA	#SI03649191	Qiagen, Hilden, Germany
5	siKLF4_2	CTGCAGCTTCACCTATCCGAT	#s17794	Ambion, Waltham, Massachusetts USA
6	siMEK5_1	AAAGGCCAGCACCTGAAGAAT	#SI00300713	Qiagen, Hilden, Germany
7	siMEK5_2	AAGACGTATGTTGGAACAAAT	#SI02222983	Qiagen, Hilden, Germany
7	siAXL_1	TCCAAGATTCTAGATGATTAA	#SI00605311	Qiagen, Hilden, Germany

Supp. Table 4: List of antibodies used

serial number	Antibody against	Species	Dilution factor	RRID	Catalgoue number	Company name
1	DUSP4	Rabbit	1:1000	AB_2750867	#5149	Cell Signalling, Frankfurt, Germany
2	ERK5	Rabbit	1:8000	AB_259274	#E1523	Sigma-Aldrich, Darmstadt, Germany
3	MEK5	Rabbit	1:1000	AB_2141263	#AB3184	Merck Millipore, Darmstadt, Germany
4	a-tubulin	Mouse	1:10000	AB_477579	#T5168	Sigma-Aldrich, Darmstadt, Germany
5	AXL	Goat	1:200	AB_354852	#AF154	R&D Systems, Minneapolis, USA
6	KLF2	Rabbit	1:1000	AB_3713633	#51221	Cell Signalling, Frankfurt, Germany
7	KLF4	Rabbit	1:1000	AB_1852541	#HPA002926	Sigma-Aldrich, Darmstadt, Germany
8	Vinculin	Mouse	1:1000	AB_477629	#V9131	Sigma-Aldrich, Darmstadt, Germany

Supp. Table 5 : List of siKLF2/4 downregulated genes

Serial Number	Gene Symbol	log2FoldChange	padj
1	*AXL*	-2,419388954	2,18763E-13
2	*ATCAY*	-1,996406512	1,85714E-07
3	*PDGFA*	-1,958544789	8,7456E-08
4	*CSDC2*	-1,736929505	2,80278E-06
5	*RHOBTB3*	-1,708904385	1,9148E-118
6	*BASP1*	-1,698896941	1,65804E-06
7	*CCL26*	-1,643524812	2,76155E-05
8	*AMZ1*	-1,633237864	2,94416E-05
9	*MAMDC2*	-1,622427277	4,6562E-09
10	*KLF4*	-1,611761046	1,01826E-24
11	*KIAA1210*	-1,583419668	6,90579E-05
12	*LOC728392*	-1,565800146	4,99685E-09
13	*LOC107984334*	-1,557649843	3,65186E-07
14	*SYNPO2L*	-1,508582507	2,06944E-07
15	*DLX3*	-1,477660123	0,000203529
16	*RASSF2*	-1,436757105	8,08602E-20
17	*FSTL1*	-1,419516588	9,09669E-08
18	*VWA2*	-1,402360634	0,000628743
19	*DKK1*	-1,401949238	3,45018E-05
20	*PDZK1*	-1,36539599	4,31809E-08
21	*TRIM67*	-1,342188546	0,000830741
22	*PLXND1*	-1,337031991	3,74949E-09
23	*SERTAD2*	-1,333963908	6,14905E-62
24	*IL12A*	-1,30412152	7,31435E-05
25	*SOAT2*	-1,282973697	0,000749686
26	*CCN5*	-1,275411121	1,21981E-27
27	*ANKRD2*	-1,273450025	0,00071493
28	*CYB5R2*	-1,252363354	8,39026E-12
29	*PKP3*	-1,233987016	0,001655721
30	*NAT16*	-1,220392582	0,00073788
31	*PDZK1P1*	-1,218695596	0,000526383
32	*ABHD2*	-1,213188935	8,05866E-86
33	*SPN*	-1,212589812	0,001823666
34	*KLF2*	-1,205406625	5,38739E-13
35	*FOLR3P1*	-1,204109241	0,002455644
36	*METRNL*	-1,197516566	2,26015E-19
37	*EID3*	-1,183410958	0,006387373
38	*IL34*	-1,177916324	0,00666047
39	*SOWAHC*	-1,176780105	1,52527E-34
40	*NPPB*	-1,174984043	0,007053834
41	*GJA5*	-1,174705601	0,000715676
42	*ELF4*	-1,173480064	6,19152E-09
43	*LOC102724908*	-1,172382867	0,006527803
44	*ARHGAP45*	-1,169433088	1,06511E-07
45	*GADD45A*	-1,157996258	1,22399E-07
46	*SH2D3C*	-1,152309411	0,000270349

47	*CLDN4*	-1,152214135	0,000708936
48	*CTSS*	-1,151438544	0,001896385
49	*TRIM54*	-1,149768589	0,008519067
50	*CORO1A*	-1,148332321	3,36781E-06
51	*H19*	-1,14611391	0,00063341
52	*LINC00707*	-1,145379471	0,007720193
53	*FLNC*	-1,145286035	1,80809E-05
54	*NKAIN3-IT1*	-1,14267204	0,008172987
55	*RASGRF1*	-1,136674215	2,78544E-06
56	*APOL3*	-1,134486281	0,000229297
57	*LOC107985992*	-1,133240203	0,009601807
58	*JDP2*	-1,131628973	0,000206174
59	*LOC102724852*	-1,129030335	0,000730007
60	*CRHBP*	-1,126796649	0,000587479
61	*BUD23*	-1,115169474	2,09607E-61
62	*CRYM*	-1,114833872	5,5512E-05
63	*SHC2*	-1,111640146	1,28646E-05
64	*LITAF*	-1,109076742	3,44701E-81
65	*JPH2*	-1,103066846	2,5049E-05
66	*CLCF1*	-1,100695881	1,80934E-08
67	*RTL3*	-1,099213666	0,000675113
68	*PSD2*	-1,095709194	0,000777364
69	*SMCO3*	-1,084724455	0,009829
70	*XAGE3*	-1,084204035	0,001582619
71	*LBR*	-1,083831165	9,27783E-31
72	*ANGPTL7*	-1,079998534	0,000461419
73	*MEX3B*	-1,076069802	9,09669E-08
74	*SLC4A8*	-1,072117044	8,48399E-06
75	*ACHE*	-1,062735745	0,000478785
76	*NTSR1*	-1,061690452	0,003858439
77	*CAMK2N2*	-1,060769033	3,49558E-09
78	*NEURL1B*	-1,056883195	1,61445E-06
79	*EGFL8*	-1,056280137	6,73758E-11
80	*LUZP6*	-1,056256425	1,90592E-88
81	*MTPN*	-1,056256425	1,90592E-88
82	*ECSCR*	-1,047263302	0,014549838
83	*C6orf120*	-1,044717274	7,62031E-71
84	*CHRM4*	-1,044518343	0,005053955
85	*LOC101927476*	-1,043438701	0,015664536
86	*S1PR5*	-1,040598253	0,012115872
87	*LYPD5*	-1,028488593	0,012458565
88	*CHRM1*	-1,02811118	6,27159E-07
89	*STYK1*	-1,025726106	0,00261009
90	*CCNA1*	-1,024884222	3,27148E-06
91	*SHF*	-1,022008319	1,01862E-16
92	*MSLN*	-1,019674952	0,02218957
93	*MAPRE1*	-1,016810371	5,7441E-121
94	*S100A2*	-1,016648444	8,17043E-06
95	*ITPKA*	-1,014896274	0,00772877
96	*JPT1*	-1,0137801	3,88311E-35

97	*MARCHF4*	-1,012433948	0,017723966
98	*WWC3*	-1,011660026	3,99783E-06
99	*OPRL1*	-1,011173012	0,000217395
100	*CD70*	-1,00980114	7,76476E-06
101	*HEG1*	-1,00837144	3,33385E-30
102	*KIF13B*	-1,004076618	2,16935E-39
103	*RBM24*	-1,003001067	0,020523503
104	*LOC388248*	-1,002235038	0,02104504
105	*CDKN1A*	-1,00046348	3,13404E-10
